# Book Reviews

**Published:** 1908-09

**Authors:** 


### BOOK REVIEWS

THE NEWER REMEDIES.—Including their Synonyms, Sources, Tests, Solubilities, Incompatibilities, Medical Properties and Doses as far as known, together with such Proprietaries as have similar titles. A reference Manual for Physicians, Pharmacists and Students. By Virgil Coblentz, A. M., Ph. M., Ph. D., F. C. S., Professor of Chemistry in Columbia University, Department of Pharmacy. Fourth Edition Revised and Enlarged. The Apothecary Publishing Co., Boston, Mass.

This work presents an alphabetical list of the newer remedies with essential facts concerning them and affords an easy and accurate method of refering to these preparations, many of which are not included in the works on materia medica, etc.

REFERENCE AND DOSE BOOK.—By. C. Henri Leonard, A. M., M. D., Emeritus Professor of Gynecology in the Detroit College of Medicine. New and enlarged edition; 40th thousand. Cloth, limp sides, round corners, thin paper, 16mo., 145 pages; price, 75 cents. The Illustrated Medical Journal Company, Publishers, Detroit, Mich.

The changes in the new edition of the U. S. Pharmacopoea are given in this edition of "Leonard's Dose Book" in two groupings, one showing those of "Increased Strength," the other of "Decreased Strength," and the new doses for these changes. All the Dose List has been carefully "proof-read" by several different readers, so as to insure absolute accuracy in the (nearly) 4,000 remedies given. The U. S. Dispensatory has been followed for medium and maximum dosage. The common name (in small type) is given after the drug name and dose. Besides this complete Dose List, the book has numerous useful Tables and a therapeutic index.

This new edition has been printed on thin paper so as to make it adaptable for buggy case or "bag," the whole being only one-fourth of an inch thick and weighing only about three ounces. Its round corners and smooth linen cover also make it "easy carrying" in the pocket. With this little book at hand you need never be at a loss for accurate dosage (new or old style) of a remedy.

THE PRINCIPLES OF PATHOLOGY.—Volume 1, General Pathology. By J. George Adami, M. A., M. D., LL. D., F. R. S., Professor of Pathology in McGill University, Montreal. Octavo, 948 pages, with 322 engravings and 16 plates. Cloth, $6.00 net. Lea & Febiger, Publishers, Philadelphia and New York, 1908.

Professor Adami's new work is one of which the whole English-speaking world of medicine may well be proud. At a bound, it reclaims for the Anglo-Saxon a territory hitherto largely conceded to the activity of the Teuton. Translations or compilations may be obtained, but the result is not to be compared with a first-hand native product in fitness for our needs. Dr. Adami stands fully on the level of his most eminent foreign confreres. He is broad and deep, but he possesses the saving quality of appreciating the fact that the easiest entrance for knowledge is by addressing the reader's faculty of reason. Having found this the best way to acquire knowledge himself, he so imparts it to others. His book is, accordingly, one that explains, connects facts in their natural relationship, rivets the attention and impresses the memory. In a clear and delightful style he displays the whole subject of General Pathology in this volume, to be followed by one on Systemic (including Special) Pathology. The prenomena of disease, or Pathology, are as definite and rationally connected as the phenomena or health, or Physiology. Obviously, the student and physician must have a thorough grasp of the underlying facts of disease, and must understand what it is in itself, before rational treatment is possible. Precisely this knowledge is available in its latest developments in Prof. Adami's pages, excellently set forth and amply embellished with illustrations.

PREVALENT DISEASES OF THE EYE.—By Samuel Theobald, M. D., Clinical Professor of Ophthalmology and Otology, Johns Hopkins University. Octavo of 551 pages, with 219 text-illustrations, and 10 colored plates. Philadelphia and London. W. B. Saunders Company, 1906. Cloth, $4.50 net; Half Morocco, $5.50 net.

With few exceptions all work on diseases of the eye, although written ostensibly for the general practitioner, are really adapted only for the specialist; but in Dr. Theobald's book *the requirements of the physician engaged in general practice have been made paramount.* For this reason only the common ocular maladies which the practioner is constantly called upon to treat, the simpler operations which he is justified in performing, and the aids to diagnosis which can but prove helpful to him are described in detail and illustrated with many practical illustrations, mostly original. The difficulties with which he will have to contend in diagnosing ocular diseases have been made to aid him along this line. In the matter of treatment his needs are again particularly considered, the directions given being clear ad concise, in every case only one definite treatment being given. Just such a work has not heretofore been written, so that *Dr. Theobald's work is the one book written particularly for the physician engaged in general practice, and we commend it to our readers.*

